# *Echinococcus granulosus* sensu stricto G1 is the predominant genotype in human and livestock isolates from Turkey and Iran, based on mitochondrial *nad*5 gene differentiation

**DOI:** 10.1186/s13071-021-04869-1

**Published:** 2021-07-20

**Authors:** Saeed Shahabi, Bahador Sarkari, Afshin Barazesh

**Affiliations:** 1 Department of Medical Entomology and Vector Control, School of Health, Shiraz University of Medical Sciences, Shiraz, Iran; 2 Department of Parasitology and Mycology, School of Medicine, Shiraz University of Medical Sciences, Shiraz, Iran; 3 Basic Sciences in Infectious Diseases Research Center, Shiraz University of Medical Sciences, Shiraz, Iran; 4 Department of Microbiology and Parasitology, Faculty of Medicine, Bushehr University of Medical Sciences, Bushehr, Iran

**Keywords:** Genotype, *Echinococcus granulosus* s.s., G1/G3, *nad*5, Turkey, Iran

## Abstract

**Background:**

Different genotypes of *Echinococcus granulosus* sensu stricto (s.s.) isolated from livestock and humans have been identified based on *cox*1 and *nad*1 genomic fragments. The present study was performed to differentiate the G1/G3 genotypes of *Echinococcus granulosus* (s.s.) isolated from humans and livestock (sheep and cattle) from Azerbaijan in northwestern Iran, Fars Province in southern Iran, and Van province in Eastern Turkey, using the *nad5* gene fragment as a suitable marker to distinguish these two genotypes.

**Methods:**

A total of 60 pathologically confirmed human hydatid cysts and 90 hydatid cyst samples from livestock were collected from Turkey and Iran. PCR was performed on all of the samples, targeting the *nad*5 gene. Based on PCR product quality, host type, and the geographical area where the samples were obtained, 36 of the samples were sequenced and were used in the phylogenetic analysis.

**Results:**

Out of 36 evaluated samples, 26 (72.2%) samples belonged to G1, and 10 (27.8%) samples belonged to the G3 genotype. Out of 21 samples from Turkey, 16 (76.2%) were G1 and 5 (23.8%) were G3, while out of 15 samples from Iran, 10 (66.7%) were G1 and 5 (33.3%) were the G3 genotype. None of the samples isolated from humans in Iran or from sheep in Turkey were G3. Overall, between the two countries, 18.18% of *E. granulosus* isolates in cattle, 41.66% of isolates in sheep, and 23.07% of human samples were identified as G3, and the others as the G1 genotype. The G3 genotype was not detected in human samples from Iran or sheep samples from Turkey.

**Conclusion:**

The findings of the study revealed that the G1 genotype of *E. granulosus* s.s. is the predominant genotype in humans and livestock, both in Turkey and Iran. The ratio of the *E. granulosus* s.s. G1 to G3 genotype was 3.2 in Turkey and 2 in Iran. The study also further confirmed that the *nad*5 gene properly differentiated the G1/G3 isolates of *E. granulosus* from both humans and livestock.

**Graphical Abstract:**

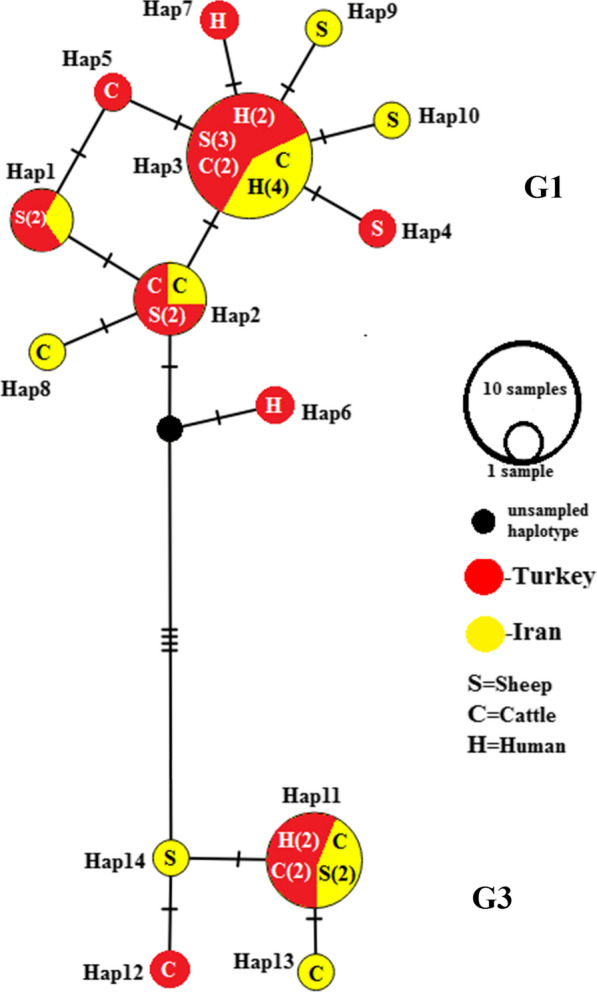

## Background

Cystic echinococcosis (CE), one of the most important zoonotic diseases, is caused by the larval stage of *Echinococcus granulosus* sensu lato (s.l.) [1]. *E. granulosus* s.l. contains a set of different genotypes that differ in life cycle patterns and host types. Currently, 10 genotypes of *E. granulosus* have been identified using different molecular methods, and are classified into four main groups: sensu stricto (genotypes G1 to G3), *equinus* (G4), *ortleppi* (G5), and *E. granulosus* s.l. genotypes (G6-G10) [2, 3]. The names for genotypes G6–G10, including *E. canadensis* and *E. intermedius*, are still under dispute, as recent molecular phylogenetic analysis based on six nuclear genes suggested that *E. granulosus* s.l. genotypes G6/G7 and G8/G10 can be regarded as two distinct species [4]. The most common hydatid cyst genotypes reported worldwide are two closely related genotypes, G1 and G3, of the sensu stricto strain [5–7].

A variety of mitochondrial genes, including *cox*1, *nad*1, and *atp*6 or a fragment sequence of the *12SrRNA* gene, as well as the internal transcribed spacer (ITS1) genomic region, have been utilized in various studies for the phylogenetic analysis of *E. granulosus* [8–12].

Regarding the G1–G3 genotype, a recent study by Kinkar et al. found that G2 is not a valid genotype [13]. Sequences of genomic fragments of *cox*1 and *nad*1 have been widely used to differentiate between G1 and G3 genotypes in various studies [14–16]. However, given the whole sequences recorded from these genomes, after several decades it has been found that these markers are not able to distinguish between these two genotypes [13]. In a recent study aimed at identifying and differentiating the G1 and G3 genotypes, a simple and practical method was introduced using a 680 bp *nad*5 genome fragment of *E. granulosus* s.s. This region contains six useful sites with relatively short lengths that allow us to properly differentiate the G1 and G3 genotypes [13].

Iran and Turkey are considered highly endemic areas for hydatid cysts in both humans and animals [6, 17–24]. In our previous studies on isolates of *E. granulosus* s.s. from livestock and human populations from Iran and Turkey, different genotypes of the parasite were identified based on *cox*1 and *nad*1 genomic fragments [5, 8]. However, since the genomic fragments used in our previous studies were not able to differentiate between the G1 and G3 genotypes, the present study was performed using specific primers and targeting the *nad*5 genomic fragment to determine the G1/G3 genotype of *E. granulosus* s.s. isolated from livestock and human samples from Azerbaijan and Fars Provinces in northwestern and southern Iran, and Van Province in eastern Turkey.

## Methods

### Study area

The study was performed on hydatid cyst samples collected from three regions: Van province from Turkey, located in the east of Van Lake, which is a part of the coldest region in Turkey; East Azerbaijan province as a cold area, located on the mountain range of Iran in the southeast of Urmia Lake; and Fars Province in southern Iran (Table 1). Fars Province has a completely different geographical and climatic condition in comparison with the other two areas in Iran and Turkey, but all three areas have always been considered endemic areas for human CE [5, 8, 24–27].

**Table 1:** Specimen voucher code (SVC), sampling location (SL), host, accession number (acc. no.), haplotype number (Hap), and genotype (G) of *nad*5 gene sequences of *E. granulosus* s.s. in human and livestock from Turkey and Iran

SVG	G	Host	Hap	SL	Acc. no.	SVG	G	Host	Hap	SL	Acc. no.
A1	G1	Sheep	H1	Turkey	MW835719	C10	G1	Human	H7	Turkey	MW835734
A2	G1	Sheep	H2	Turkey	MW835720	C13	G3	Human	H11	Turkey	MW835749
A3	G1	Sheep	H3	Turkey	MW835721	C14	G3	Human	H11	Turkey	MW835750
A4	G1	Sheep	H1	Turkey	MW835722	C16	G1	Human	H3	Iran	MW835735
A5	G1	Sheep	H2	Turkey	MW835724	C17	G1	Human	H3	Iran	MW835736
A11	G1	Sheep	H4	Turkey	MW835725	C21	G1	Human	H3	Iran	MW835737
A12	G1	Sheep	H3	Turkey	MW835723	C27	G1	Human	H3	Iran	MW835738
A9	G1	Sheep	H3	Turkey	MW835726	C28	G1	Human	H1	Iran	MW835739
B1	G1	Cattle	H3	Turkey	MW835727	CF1	G3	Cattle	H11	Iran	MW835751
B2	G1	Cattle	H3	Turkey	MW835728	CF3	G3	Cattle	H13	Iran	MW835752
B4	G3	Cattle	H12	Turkey	MW835746	CF6	G1	Cattle	H3	Iran	MW835740
B5	G3	Cattle	H11	Turkey	MW835747	CF7	G1	Cattle	H8	Iran	MW835741
B7	G3	Cattle	H11	Turkey	MW835748	CF10	G1	Cattle	B2	Iran	MW835742
B9	G1	Cattle	H5	Turkey	MW835729	S7	G1	Sheep	H9	Iran	MW835743
B10	G1	Cattle	H2	Turkey	MW835730	S9	G1	Sheep	H10	Iran	MW835744
C1	G1	Human	H3	Turkey	MW835731	S13	G3	Sheep	H11	Iran	MW835754
C3	G1	Human	H3	Turkey	MW835732	S15	G3	Sheep	H11	Iran	MW835745
C7	G1	Human	H6	Turkey	MW835733	S4	G3	Sheep	C14	Iran	MW835753

### Sample preparation

Human and livestock isolated hydatid cysts collected from Turkey and Iran in previous studies, which were identified as G1/G3 genotypes by targeting *cox*1 and *nad*1 regions of mitochondrial genome fragments, were re-evaluated by molecular methods. A total of 60 human hydatid cysts, surgically removed and pathologically confirmed, from populations in Tabriz (East Azerbaijan Province, in the northwest of Iran), Shiraz (Fars Province, southern Iran), and Van Province (20 samples from each center), and 90 hydatid cysts from livestock (30 samples from each center) were evaluated in the current study.

### DNA extraction

DNA was extracted from each sample as previously described [8]. Briefly, 100 μL of *E. granulosus* s.s. protoscolices and 25 mg of germinal layers were prepared in different microtubes. A lysis buffer and proteinase K were added to the samples and incubated for 2 h at 60 °C followed by overnight incubation at 37 °C. The rest of the procedure was performed as previously described [8].

### Polymerase chain reaction (PCR) and DNA sequencing

Amplification of the *nad*5 gene was carried out using the two primers EGnd5F1 and EGnd5R1 [28]. For PCR amplification, a final volume of 25 µL reaction containing 3 µL of extracted DNA, 1 µL (10 pm) of each primer, 12.5 µL of 1× Taq DNA Polymerase Master Mix RED (Ampliqon, Odense, Denmark), and 8.5 µL of distilled water (DW) was used. The thermal PCR conditions consisted of initial denaturation at 95 °C for 5 min, and a touchdown protocol with 10 cycles of 95 °C for 20 s, 55 °C for 45 s (annealing temperature progressively reduced by 0.5 °C in each cycle), and 68 °C for 1 min, followed by 25 cycles of 95 °C for 30 s, 50 °C for 45 s, 68 °C for 1 min; finishing with a final elongation step at 68 °C for 5 min. Individual PCR products (2 μL) were examined on 2% agarose gel electrophoresis. PCR was performed on 90 cyst samples (considering the host origin and geographical region where the samples were obtained), targeting the *nad*5 fragment. Of which, 46 PCR products were selected in terms of the quality of the resulting band on the electrophoresis gel and were sequenced in both directions using the same primers used in the PCR. Out of 46 obtained sequences, the sequences of 36 samples (21 samples from Turkey and 15 samples from Iran, including 11 human samples, 13 sheep, and 12 cattle samples), were of high quality and were used in the phylogenetic analysis.

### Phylogenetic and genetic analysis

A total of 36 raw nucleotide sequences were reviewed and analyzed. Consensus sequences were assembled and multiple-aligned with a set of *E. granulosus* s.s. strains retrieved from the GenBank database. The final aligned sequences with a total of 631 positions were converted in FASTA and MEGA format for further analysis using MEGAX software [29]. The number of base substitutions per site from averaging over all sequence pairs between groups (Kimura 2-parameter genetic distance) was computed using the Kimura 2-parameter model. Codon positions included were 1st + 2nd + 3rd + Noncoding. All ambiguous positions were removed for each sequence pair (pairwise deletion option). The best DNA substitution model of HKY+ (- lnL = 1365.6259, *k* = 89, gamma shape = 0.2 p-inv = 0.6850, AIC = 2909.2517, BIC = 3305.062, kappa = 42.3304 (*ti*/*tv* = 15.3337), freq *A* = 0.1611, freq* C* = 0.0962, freq *G* = 0.2563, and freq *T* = 0.4864) was identified using both the Akaike (AIC) and Bayesian (BIC) information criterion using jModelTest, version0.1.1 [30].

Phylogenetic relationships were reconstructed using a Bayesian inference (BI) tree in MrBayes version 3.1.2, with parameters estimated as part of the analysis. A haplotype network of *nad*5 data was constructed using the median-joining approach available in PopArt [31]. Genetic diversity based on haplotype diversity (Hd) and nucleotide diversity (π) as well as the number of polymorphic and parsimony-informative sites were measured using DnaSP v5.0 software. The sequences with accession numbers MK682655-56 and MK682657-58 [32] were used in the phylogenetic analysis as references for the G1 and G3 genotypes, respectively. The genotypes G5 (Acc. AB235846) [33], G7 (Acc. MK682636) [32], and G6 (Acc. MH300946) [34] were used as an outgroup for reconstructing the molecular phylogenetic tree.

## Results and discussion

The most common genotypes of *E. granulosus* s.s. causing hydatid cysts in humans and animals are two closely related genotypes, G1 and G3 [16, 35, 36]. For molecular evaluation and phylogenetic analysis of *E. granulosus* in very close species, different mitochondrial and nuclear genomes have been used. In the present study, the *nad*5 genomic fragment was used to differentiate between G1 and G3 genotypes in hydatid cysts isolated from humans and livestock in Iran and Turkey.

PCR products of 36 hydatid cyst samples, including 21 samples from Turkey and 15 samples from Iran, were sequenced for the *nad*5 gene. The sequences were deposited in the GenBank database (NCBI) with related accession numbers. Table 1 shows the sample’s location, the respective host, the parasite genotype, and the assigned accession number in GenBank.

Out of 36 evaluated samples, 26 (72.2%) samples belonged to G1 and 10 (27.8%) samples belonged to the G3 genotype. Out of 21 samples prepared from Turkey, 16 (76.2%) were G1 and 5 (23.8%) were G3, while out of 15 samples prepared from Iran, 10 (66.7%) were G1 and 5 (33.3%) were G3. Overall, in both countries, 18.18% of *E. granulosus* isolates in cattle, 41.66% of isolates in sheep, and 23.07% of human samples were identified as G3 and others as G1 genotype. Table 2 shows the number of G1/G3 genotypes isolated from different hosts in Iran and Turkey. As the data in Table 2 show, the G3 genotype was not detected in human samples from Iran or sheep samples from Turkey.

**Table 2:** Frequency of the G1/G3 genotypes of *E. granulosus* s.s. isolated from different hosts in Iran and Turkey

Host	Sheep		Cattle		Human	
Region	G1	G3	G1	G3	G1	G3
Van Province, Turkey	8	–	4	3	4	2
Fars Province, Iran	1	2	1	1	5	–
East Azerbaijan, Iran	1	1	2	1	–	–
Total in Iran	2	3	3	2	5	–

**Fig. 1:**
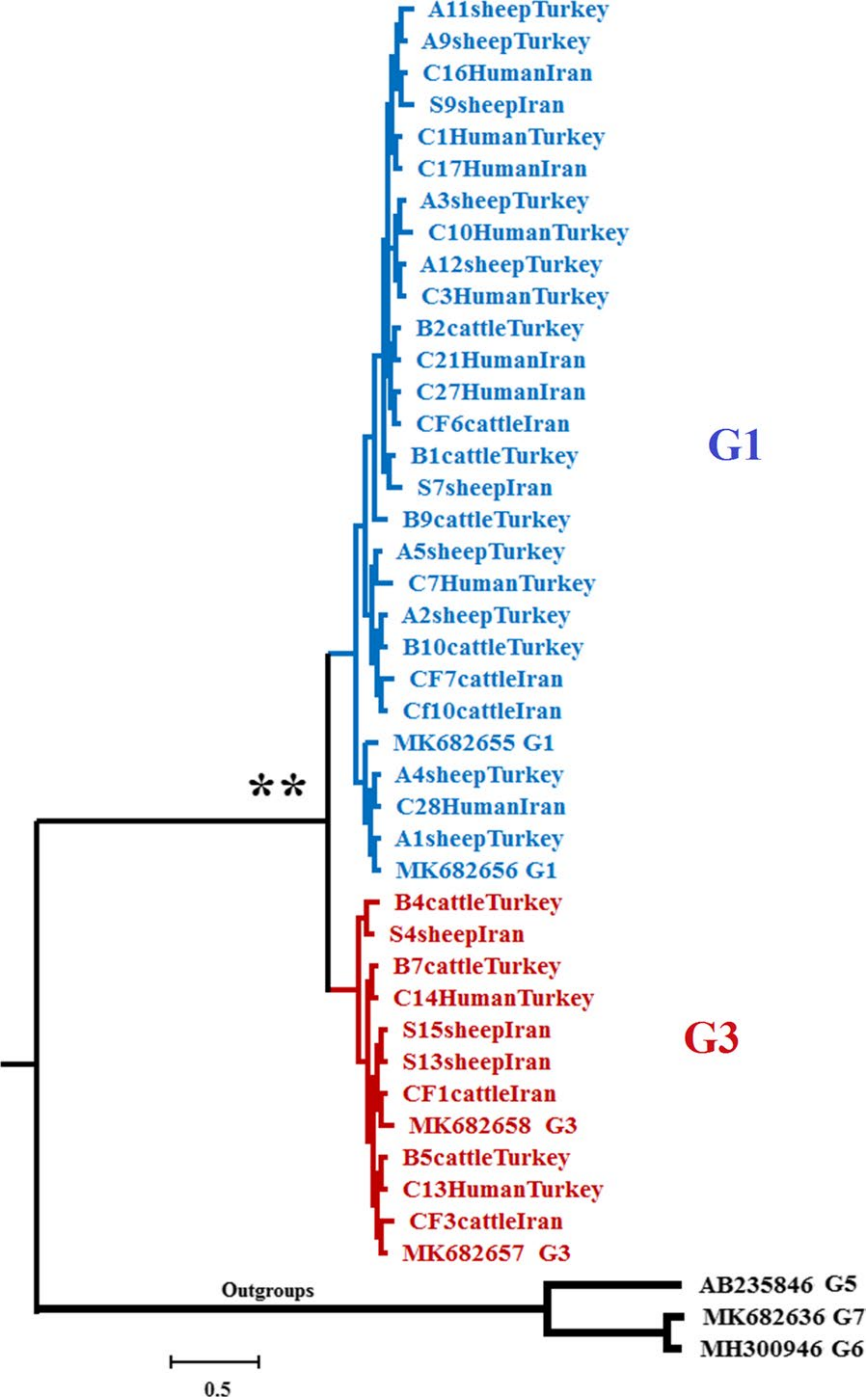
Bayesian phylogenetic tree inferred from 40 *nad*5 sequences of *Echinococcus granulosus* sensu stricto (s.s.)*.* Posterior probability values from the Bayesian analysis are indicated at the 100% (**) significance levels. Specimen name includes sample name, host, and country (Table 1)

Two highly supported major clades were recovered from the BI analysis (Fig. 1). Clade 1 comprises the G1 genotype, infecting humans and livestock from Iran and Turkey, while clade 2 includes the G3 genotype infecting humans and cattle from Turkey and livestock from Iran**.**

**Fig. 2:**
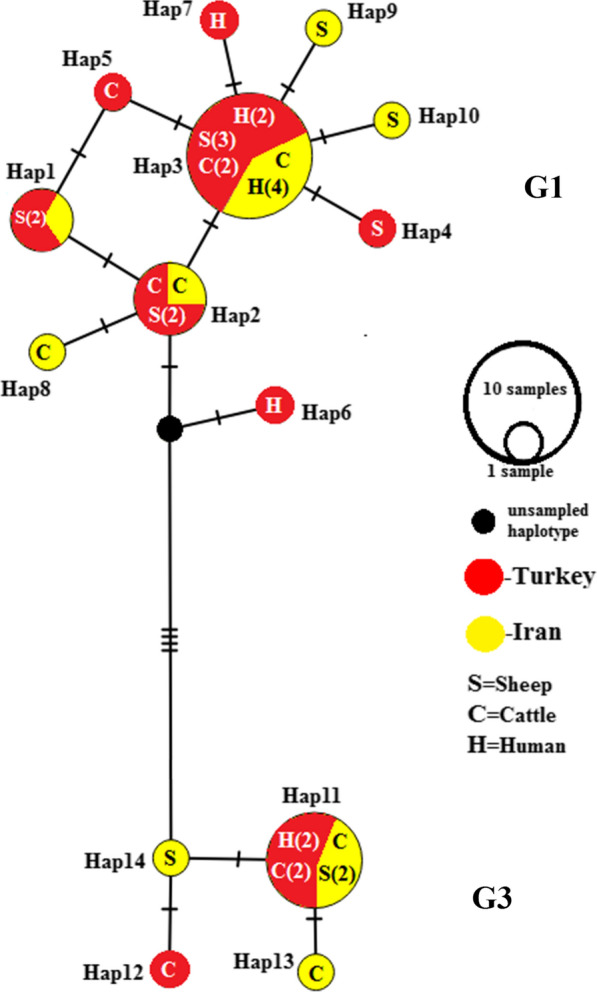
Median-joining haplotype network obtained for 631 bp of mitochondrial *nad*5 sequences of *E. granulosus* s.s. Circle size is relative to haplotype frequency; black circles represent extinct or unsampled haplotypes. Hatch marks on the line represent mutational steps between haplotypes. Haplotype colors represent geographic locations of haplotypes as indicated in the right corner of the figure (yellow = Iran, red = Turkey). Host species are indicated with letters inside the haplotypes (C = cattle, S = sheep, H = human). The numbers in parentheses inside haplotypes (in front of letters) indicate the frequency of the haplotype observed in the related host. Haplotype information is summarized in Table 1

Based on the 631 bp of the *nad*5 fragment, examined in all 36 sequences, 15 sites were polymorphic, including eight singleton variable sites (two variants) and seven parsimony-informative sites (two variants), resulting in the identification of 14 haplotypes, including 10 haplotypes in G1 and 4 in G3 genotype. Of the 14 haplotypes, 10 distinct haplotypes were observed in the G1 haplogroup from which three were shared between Iranian and Turkish isolates and four were specific to Turkey and three to Iran (Fig. 2). One haplotype (haplotype 3) in the G1 subnetwork with a frequency of 12 was the most frequent haplotype shared between Turkish livestock (3 sheep and 2 cattle), two human samples from Turkey, and four human samples from Iran (Fig. 2). The average number of nucleotide differences and nucleotide divergence between the two main clades G1 and G3 was 6/754 and 1%, respectively. The percentage of the Kimura 2-parameter (K2P) mean genetic distance between G1 and G3 was 1.08%. The percentage of mean K2P distance within the G1 and G3 groups was 0.2% and 0.12%, respectively.

Haplotype diversity (Hd) and nucleotide diversity (π) values were 0.769 and 0.002 in G1 sequences, respectively, while these values were 0.533 and 0.0012 in G3 sequences, respectively.

Haplotypes 8, 7, and 5 of *nad*5 were identified in sheep, cattle, and humans, respectively (Fig. 2). No haplotype in the G1 subnetwork was shared between Iranian and Turkish livestock, but haplotype 3 was shared between sheep and cattle from Turkey (Fig. 2). In the G3 subnetwork, haplotype 11 was shared between Iranian livestock and cattle, and two human samples from Turkey (Fig. 2).

In a study by Romig et al., a low level of differentiation was found between G1 and G3 in the haplotype network, and in a significant proportion of the samples, no distinction was made between the two genotypes [16]. In order to identify and differentiate the G1 and G3 genotypes, Kinkar et al. used data from more than 300 sequences of the parasite's mitochondrial genome to determine the genetic diversity of the two genotypes on a large geographical scale, and introduced a simple new method by sequencing the 680 bp *nad*5 genome fragment for differentiation of these two genotypes.

Concerning the significance of differentiating G1 and G3, although nuclear gene sequencing data seem to suggest that G1 and G3 form one species, these genotypes have rather different distribution and host range, which provides a good basis to at least suspect some biological relevance. In the phylogeography of many species, including the *E. granulosus* species complex, even small haplogroups can be of significance in advancing our knowledge. Differentiating G1 and G3 can also potentially help to reveal the geographical origin of the parasite in patients diagnosed with CE.

The present study is a continuation of our previous studies and is designed to highlight the prevalence of *Echinococcus* genotypes 1 and 3 in Iran and Turkey. The findings of the current study highlight the molecular epidemiology aspect of G1/G3 genotypes in two CE-endemic areas, Iran and Turkey. In addition, with the identification of the G1 and G3 genotypes of *Echinococcus*, phylogenetic analysis of these two genotypes was documented.

## Conclusion

Findings of the current study revealed that the G1 genotype of *E. granulosus* s.s. is the predominant genotype in humans and livestock in both Turkey and Iran. The ratio of the *E. granulosus* s.s. G1 to G3 genotype was 3.2 in Turkey and 2 in Iran. The study also further confirms that the *nad*5 gene properly differentiates the G1/G3 isolates of *E. granulosus* from both humans and livestock.

## Data Availability

Data supporting the conclusions of this article are included within the article. Sequences were deposited in the GenBank database (NCBI) with Accession Numbers MW835719 to 54.

## Abbreviations

CE: Cystic echinococcosis
ITS1: Internal transcribed spacer
BI: Bayesian inference
